# *HLA-*associated susceptibility to childhood B-cell precursor ALL: definition and role of *HLA-DPB1* supertypes

**DOI:** 10.1038/sj.bjc.6604257

**Published:** 2008-03-11

**Authors:** G M Taylor, A Hussain, T J Lightfoot, J M Birch, T O B Eden, M F Greaves

**Affiliations:** 1 Cancer Immunogenetics Laboratory, School of Cancer Sciences, University of Manchester, Manchester, UK; 2 Epidemiology and Genetics Unit, Department of Health Sciences, University of York, York, UK; 3 CRUK Paediatric and Familial Cancer Research Group, School of Cancer Sciences, University of Manchester, Manchester, UK; 4 Academic Unit of Paediatric and Adolescent Oncology, School of Cancer Sciences, University of Manchester, Manchester, UK; 5 Leukaemia Research Fund Cell Biology Unit, Institute for Cancer Research, London, UK

**Keywords:** *HLA-DPB1*, supertypes, BCP ALL, case–control comparison, allele frequency, peptide-binding pockets

## Abstract

Childhood B-cell precursor (BCP) ALL is thought to be caused by a delayed immune response to an unidentified postnatal infection. An association between BCP ALL and *HLA class II* (*DR*, *DQ*, *DP*) alleles could provide further clues to the identity of the infection, since HLA molecules exhibit allotype-restricted binding of infection-derived antigenic peptides. We clustered >30 *HLA-DPB1* alleles into six predicted peptide-binding supertypes (*DP1*, *2*, *3*, *4*, *6*, and *8*), based on amino acid di-morphisms at positions 11 (G/L), 69 (E/K), and 84 (G/D) of the DP*β*_1_ domain. We found that the DP*β*11-69-84 supertype GEG (*DP2*), was 70% more frequent in BCP ALL (*n*=687; *P*<10^−4^), and 98% more frequent in cases diagnosed between 3 and 6 years (*P*<10^−4^), but not <3 or >6 years, than in controls. Only one of 21 possible *DPB1* supergenotypes, GEG/GKG (*DP2/DP4*) was significantly more frequent in BCP ALL (*P*=0.00004) than controls. These results suggest that susceptibility to BCP ALL is associated with the *DP2* supertype, which is predicted to bind peptides with positively charged, nonpolar aromatic residues at the P4 position, and hydrophobic residues at the P1 and P6 positions. Studies of peptide binding by *DP2* alleles could help to identify infection(s) carrying these peptides.

Acute lymphoblastic leukaemia (ALL) is the most common childhood malignancy in developed countries, where it constitutes over 30% of childhood cancers (Stiller *et al*, 1998; Smith *et al*, 1999). The striking age-incidence peak between 2 and 5 years of age consists mainly of common, B-cell precursor (BCP) ALL (Greaves *et al*, 1993, 1985; McKinney *et al*, 1993; Buckley *et al*, 1994). Molecular data indicate that BCP ALL can arise *in utero* in association with acquired chromosomal rearrangements that result in covert preleukaemic clones (Wiemels *et al*, 1999; Greaves, 2006), but progression to clinical ALL requires additional clonal genetic abnormalities, accumulated in a variable postnatal latent period. These may arise under the influence of an immune response to delayed infection (McNally and Eden, 2004; Greaves, 2006), but lack of information incriminating a specific infectious agent (Greaves, 2006; MacKenzie *et al*, 2006) has hindered verification of this causal pathway.

Insights into the role of infection in the aetiology of BCP ALL could be provided by associations with *HLA class II* alleles (Dorak *et al*, 1995, 1999; Taylor *et al*, 1995, 1998, 2002). The highly polymorphic *HLA DR*, *DQ*, and *DP* loci are encoded by genes in the human major histocompatibility complex (MHC), and are responsible for the binding and presentation of infection-derived peptides to CD4+ T cells, leading to adaptive immune responses to infections (Cooke and Hill, 2001). The affinity of different HLA class II allotypes for infection-derived peptides is influenced by a series of discrete peptide-binding pockets (PBP) embedded in the antigen-binding groove of the HLA class II *α*/*β* heterodimer (Hammer *et al*, 1997).

Since T-cell responses to infection in the presymptomatic phase of BCP ALL are not readily accessible to functional analysis, *HLA class II* alleles provide a potential PBP ‘footprint’ of the infection that may be involved in this disease. However, tight linkage between the *HLA-DR* and *DQ* loci makes it difficult to distinguish the primary contribution of alleles at these loci. Contrasting patterns of *DR-DQ* allelic linkage disequilibrium (LD) in different ethnic groups (Oksenberg *et al*, 2004) could resolve this problem, but such studies have yet to be reported in childhood leukaemia. Since the *HLA-DP* locus is only weakly linked to *DR-DQ* (Begovich *et al*, 1992; Cullen *et al*, 2002), analysis of *DP* alleles in BCP ALL should identify associations independent of *DR-DQ*. We and others have previously reported associations between *DP* alleles and human leukaemia (Pawelec *et al*, 1988; Taylor *et al*, 1995, 2002). Furthermore, *DP* alleles are known to be associated with, or to act as restriction elements for a number of parasitic (Meyer *et al*, 1994; May *et al*, 1998), microbial and viral diseases, including hepatitis B and rabies (Celis and Karr, 1989; Celis *et al*, 1990), herpes simplex (Koelle *et al*, 2000), streptococcus (Dong *et al*, 1995), dengue virus (Kurane *et al*, 1993; Okamoto *et al*, 1998), Epstein–Barr virus (Voo *et al*, 2002), respiratory syncytial virus (RSV) (De Graaf *et al*, 2004; De Waal *et al*, 2004), and HIV (Cohen *et al*, 2006).

Peptide binding by HLA class II allotypes, including DP, is the outcome of interactions between the amino acid side chains of the peptide and four major peptide-binding pockets (1, 4, 6, and 9; Hammer *et al*, 1997). Since different alleles can have overlapping peptide-binding properties, depending on the number of PBP that they share (Southwood *et al*, 1998), this has permitted *DR* alleles with the same amino acid polymorphisms lining specific peptide-binding pockets to be clustered into supertypes (Sette and Sidney, 1998; Southwood *et al*, 1998; Doytchinova and Flower, 2005). Using a similar approach, Castelli *et al* (2002) defined three *DP* supertype clusters with shared amino acid residues in the P1 (*β*84) and P6 (*β*11) PBP. However, the P4 peptide-binding pocket, at position *β*69, also makes an important contribution to antibody and peptide-binding (Arroyo *et al*, 1995; Chicz *et al*, 1997), T-cell responses (Berretta *et al*, 2003; Diaz *et al*, 2003) and disease susceptibility (Potolicchio *et al*, 1999; Wang *et al*, 1999). For this reason we clustered >30 *DPB1* alleles into six supertypes based on polymorphisms in three PBP, at positions 11, 69, and 84 of the *β*1 domain (i.e., pockets 6, 4, and 1). We compared their frequencies in childhood BCP ALL, non-BCP leukaemia and solid tumours recruited as part of the UK Childhood Cancer Study (2000) with newborn controls. We discuss the implications of our findings in relation to an infectious aetiology for BCP ALL.

## MATERIALS AND METHODS

### Cases and controls

Childhood leukaemia cases were recruited between 1992 and 98 as part of the UK Childhood Cancer Study (UKCCS, 2000). Leukaemias were classified as BCP ALL (CD10+, CD19+; *n*=687) or non-BCP acute leukaemia. The non-BCP leukaemias were the sum of Pro-B ALL (CD10−, CD19+), T-ALL (CD2/CD7+, CD19−, DR−), and AML (*n*=208). Diagnostic immunophenotyping was carried out according to the protocol for UK Medical Research Council leukaemia trials (UKCCS, 2000). Childhood solid tumour cases (*n*=409) were also recruited as part of the UKCCS (UKCCS, 2000). Umbilical cord blood samples from a cross-sectional series of normal white UK newborns (*n*=864) born in Manchester UK between 1991 and 1997 were used as controls (Taylor *et al*, 2002). Blood sample collection and *HLA* molecular typing were carried out with national and local ethical consent. UKCCS patient data (diagnoses, gender, ages, ethnic background) were validated by the UKCCS data centre at the Epidemiology and Genetics Unit, University of York.

### *HLA-DPB1* molecular typing

*HLA-DPB1* molecular typing was carried out as previously described in detail (Taylor *et al*, 2002) by amplifying a 327 bp exon 2 product in each case and control genomic DNA sample using a single pair of generic *DPB1* PCR primers, spotting aliquots of each PCR product onto 384 sample nylon filters, and hybridising replicate filters with a panel of 28 ^32^P-labelled sequence specific oligonucleotide probes. Probe hybridisation was detected using real-time autoradiography, and alleles assigned from published *DPB1* ideograms.

### Data analysis

*DPB1* alleles in cases and controls were grouped into the six supertype clusters defined in this study (see Table 2 and Results for further details). Supertype allele and genotype frequencies were compared in cases and controls using global and univariate statistical analysis. As discussed previously (Taylor *et al*, 2002) ethical constraints precluded the collection of samples from case-matched control children, so we used local white UK newborns as controls. *DPB1* alleles with a cumulative frequency of <5% that did not fall within the supertype clustering system were excluded from the analysis. Only sequence variation in the three peptide-binding pockets (positions 11, 69, and 84; pockets 6, 4, and 1, respectively) used for supertype clustering was included in the analysis. Global case–control supertype frequencies were compared using the CLUMP programme of Sham and Curtis (1995), a Monte Carlo method that computes a Pearson *χ*^2^ statistic (T1) from a series of simulated case–control tables. In univariate analysis, cross-product odds ratios (ORs), and 95% confidence intervals were calculated from case–control supertype and genotype frequencies by the RERI program in the Linkage Utility Package, LINKUTIL, using the Sheehe method. The 2by2 programme in LINKUTIL was used to determine 2-sided *P*-values for case–control supertype and genotype differences using Fisher's Exact test. Six supertypes require an uncorrected *P*-value <0.008, and 21 supergenotypes an uncorrected value <0.002 to achieve significance (*P*=0.05). No correction for the total number of classical *DP* alleles was applied. POPGENE version 1.31 was used to test for two-locus linkage disequilibrium between *DPB1* and *DQA1*, or *DQB1* alleles.

## RESULTS

### Case and control characteristics

The UKCCS is an epidemiological case–control study designed to test the role of environmental factors in the aetiology of childhood cancer and leukaemia (UKCCS, 2000). As part of the UKCCS, we obtained *HLA-DPB1* types for 982 cases of childhood leukaemia (Taylor *et al*, 2002). Ninety-one percent of the leukaemia cases were classified as white, based on parental information, the remainder being Asian (3.8%), Black (1%), mixed ethnicity (1.9%), other ethnic groups (0.5%) or unknown. Of 875 cases of ALL, 559 were identified as BCP ALL, and a further 228 ALL cases were unclassified (Taylor *et al*, 2002). Subsequent diagnostic information for the unclassified ALL cases enabled us to identify 128 additional BCP ALL, seven Pro-B ALL, and six T ALL cases. These were included in the present study, which therefore comprises 895 *DP*-typed cases of childhood leukaemia with a confirmed diagnosis, of which 687 were BCP ALL and 208 were non-BCP leukaemia cases (Table 1). A mixed diagnostic series of childhood solid tumour cases (*n*=409), not including childhood lymphoma (Taylor *et al*, 2002) is included for comparison. Of these, 405 cases had informative ethnic data, being classified as white in 91% of cases. Cord blood samples from a cross-sectional series of normal white UK term newborns (*n*=864) were used as controls. Male–female ratios were slightly higher in the leukaemia cases (1.22) than the solid tumours (1.14) and controls (1.01).

### *HLA-DPB1* supertypes

The majority (90%) of >30 *DPB1* alleles in the cases and controls could be clustered into six supertypes (Table 2), consisting of three pairs, each pair differing at position 69 for a glutamic acid (E) or lysine (K) in pocket 4, but having the same residues at positions 11 (G or L; pocket 6) and 84 (G or D; pocket 1). We designated the six supertypes by their position 11-69-84 residues as GEG, GKG, LED, LKD, GED, and GKD, corresponding to dimorphisms in the P6-P4-P1 peptide-binding pockets. Using a modification of the hierarchical supertype clustering system for *DP* alleles developed by Doytchinova and Flower (2005), we have provisionally called these supertypes *DP1* (GKD), *DP2* (GEG), *DP3* (LKD), *DP4* (GKG), *DP6* (LED), and *DP8* (GED).

### *HLA-DPB1* supertype frequency in childhood leukaemia

In the total leukaemia case series (*n*=895) and the newborn controls (*n*=864), we identified 14 *DPβ69E* alleles, of which four are *DP2* (GEG), seven are *DP6* (LED), and three are *DP8* (GED). Of 15 *DPβ69K* alleles, six are *DP4* (GKG), six are *DP3* (LKD), and three are *DP1* (GKD). In global *χ*^2^ analysis, the supertype frequency in the total leukaemia series was significantly different (*P*<10^−6^) from the controls (Table 3), but there was only a marginal difference between the solid tumour cases and controls (*P*=0.04). In univariate analysis, *DP2* (GEG) (OR, 95% confidence interval (CI): 1.6, 95%, CI, 1.2–2.0; 2 sided *P*=0.0002) and *DP8* (GED) (OR, CI: 2.9, 1.4–6.3; *P*=0.006) were significantly more frequent in leukaemia cases than controls. *DP6* (LED) (OR, CI: 1.3, 1.0–1.8; *P*=0.04) was only marginally significant without correction for six supertypes, while *DP2* and *DP8* were significant after correction.

Stratification of the leukaemias into BCP ALL (*n*=687) and non-BCP acute leukaemia (*n*=208) revealed that *DP* supertypes in BCP ALL differed significantly from the newborn controls (*P*<10^−6^), but non-BCP leukaemia was only marginally significant (*P*=0.04) (Table 4). In univariate analysis, *DP2* (GEG) (OR, CI: 1.7, 1.3–2.1; *P*<10^−4^) and *DP8* (GED) (OR, CI: 3.2, 1.5–7.0; *P*=0.004) were significantly more frequent, after correction for six supertypes, than controls. *DP6* (LED) was not significant in BCP ALL, but was significant in non-BCP leukaemia (OR, CI: 1.8, 1.2–2.7; *P*=0.007). *DP1* (GKD) was significantly less frequent, after correction, than controls in BCP ALL (OR, CI: 0.5, 0.4–0.7; *P*<10^−5^), but not in non-BCP leukaemia.

The association of BCP ALL with *DP2* and *DP8* raised the possibility of a chance finding. To test this, supertype frequencies in four BCP ALL case series were compared with controls: (1) cases included in our previous study (*n*=559; Taylor *et al*, 2002); (2) half of the cases in the present study (*n*=344); (3) half of the cases in the previous study combined with the ‘new’ cases (*n*=343); (4) the ‘new’ cases (*n*=128) alone. *DP2* and *DP8* were significant in all four case series, though only *DP2* remained significant after correction (Table 5).

**Figure 1:**
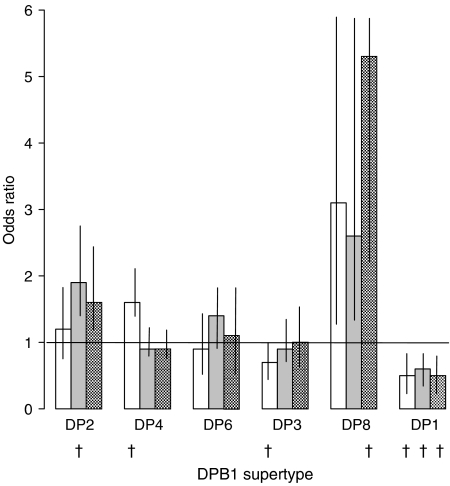
Odds ratios for *DPB1* supertype frequencies compared with normal newborns in relation to the age at diagnosis of BCP-ALL. Ages at diagnosis: 0–<3 years (white bar), 3–6 years (grey bar), >6 years (checked bar). Vertical limits are 95% confidence intervals. ^†^One-sided, corrected Fishers *P*-values: 0–<3 years: *DP4*=0.018, *DP3*=0.012; *DP1*=0.012. 3–6 years: *DP2*=0.0006, *DP1*=0.012. >6 years: *DP8*=0.018; *DP1*=0.03.

To determine the relationship between the age at diagnosis of BCP ALL and *DP* supertype, we compared the frequencies in cases diagnosed <3 years of age, >3–6 years, and >6 years, with controls. Figure 1 shows that the risk of BCP ALL was increased by 98% in *DP2*+ cases diagnosed at >3–6 years (OR, CI: 1.9, 1.4–2.6; *P*=10^−4^), but was not significant in BCP ALL diagnosed <3 or >6 years. *DP4* was significantly increased in BCP ALL diagnosed <3 years, though not after correction. *DP8* was not significant after correction, while *DP1* protected from BCP ALL in all age groups.

### *HLA-DPB1* supergenotype frequency

To determine which combination of supertype alleles was associated with BCP ALL, we compared the frequency of all 21 possible supergenotypes (six homozygous, 15 heterozygous) in BCP ALL, non-BCP leukaemia, and solid tumours with newborn controls (Table 6). Note that certain heterozygous *DPB1* genotypes, such as DPB1*0201/0202 can have a homozygous supergenotype (*DP2/DP2*:GEG/GEG) (Table 2). Of the 21 supergenotypes, only one (*DP2/DP4*), was associated with a significantly increased risk (110%), after correction, of BCP ALL (OR, CI: 2.1, 1.5–2.9; *P*=0.00004). *DP2/DP4* was associated with an increased risk (130%) of BCP ALL arising between 3 and 6 years of age (OR, 95% CI: 2.3, 1.4–3.8; *P*=0.04), but not <3 or >6 years of age. No *DP2* supergenotypes were significant in non-BCP leukaemia or solid tumours. *DP4/DP6* (GKG/LED) was significant in non-BCP leukaemia after correction (OR, CI: 2.7, 1.6–4.9; *P*=0.002), but not in BCP ALL or solid tumours. Homozygous *DP1* (GKD/GKD) significantly protected, after correction, against BCP ALL (OR, CI: 0.2, 0.1–0.5; *P*=0.00004). No other *DPB1* supergenotypes were significant at any of the diagnostic ages.

### Linkage disequilibrium analysis

To test whether the *DP* supertype associations could be explained by LD with *HLA-DQ* alleles, we analysed the co-occurrence of *DP and DQ* alleles in 451 BCP ALL cases, using POPGENE. We detected only one *DP* allele, *1601*, in LD with *DQ* (DQB1*0401; *χ*^2^=37.4; uncorrected *P*<10^−4^). Five BCP ALL cases (0.4%) typed for DPB1*1601, a frequency not significantly greater than in the controls, indicating that the *DP*-supertype results cannot be explained by LD between *DP* and *DQ* alleles.

## DISCUSSION

Selective peptide binding by HLA allotypes is a prerequisite for the recognition of antigens by T cells leading to adaptive immunity (Madden, 1995). Such a mechanism may underpin the immune-mediated progression of pre-ALL to overt leukaemia following delayed postnatal infection (Greaves, 2006). In our previous study, we suggested that the presence in pocket 4 of a glutamic acid (E) residue at position 69 of the DP*β*1 domain was associated with BCP ALL (Taylor *et al*, 2002). However, HLA class II allotype-associated peptide binding is not the property of a single PBP; rather, it is the sum of a series of key PBP forming a DP allotype-associated peptide-binding motif or ‘footprint’. Polymorphisms in PBP accommodating the P1, 4, 6, and 9 amino acid anchors appear primarily to influence the DP allotype footprint (Hammer *et al*, 1997; Diaz *et al*, 2003, 2005). Since pocket 9 is composed of polymorphisms in residues 9, 35, 36, 55, and 56 (Diaz *et al*, 2003), we excluded this level of complexity. Furthermore, grouping amino-acid polymorphisms at positions 36, 56, and 76 failed to define recognised supertypes, and were not associated with leukaemia (data not shown). Clustering of *DP* alleles into six supertypes based on amino acid dimorphisms at positions 84 (P1 pocket), 69 (P4 pocket), and 11 (P6 pocket) represents an expanded version of the scheme proposed by Castelli *et al* (2002) based on peptide binding, and a slightly modified version of the hierarchical clustering scheme proposed by Doytchinova and Flower (2005). We have provisionally denoted the six supertypes *DP1* (GKD), *DP2* (GEG), *DP3* (LKD), *DP4* (GKG), *DP6* (LED), and *DP8* (GED) since they broadly resemble those defined in the primed lymphocyte test (PLT) as *DPw* specificites (De Koster *et al*, 1991). Furthermore, *HLA-DPw2* defined by PLT was previously reported to be associated with ALL (Pawelec *et al*, 1988).

The *DPB1* locus is the second most polymorphic *HLA class II* locus after *DRB1*, with at least 120 alleles identified to date (http://anthonynolan.org.uk/HIG/lists/class2list.html). In a rare disease such as BCP ALL in which there are likely to be multiple aetiological factors, weak *HLA* associations potentially require hundreds of cases and controls to allow for correction for multiple testing. Supertype analysis, in which alleles are clustered according to common functional (i.e., peptide binding) properties, overcomes this problem. *DPB1* alleles comprise combinatorial series of six variable regions (A-F) encoded by exon 2 (Bugawan *et al*, 1988), in which alleles with the same variable region polymorphisms have the same peptide-binding pockets. *DP* alleles with the same polymorphisms at position 11 in variable region A, position 69 in variable region D, and position 84 in variable region F (Bugawan *et al*, 1988) can be predicted to have similar immune functions, based on identical (P6, P4, and P1, respectively) PBP. Our supertype classification includes position 69 (P4 pocket) since this is known to influence antibody-binding (Arroyo *et al*, 1995), allorecognition and peptide binding (Diaz *et al*, 2005), and disease susceptibility (Potolicchio *et al*, 1999; Wang *et al*, 1999). Furthermore it allowed us to split *β69E* alleles into three supertypes (GEG (*DP2*), LED (*DP6*), GED (*DP8*)), and to compare these with three homologous *β69K* series (GKG (*DP4*), LKD (*DP3*), GKD (*DP1*)).

We observed a 70% increase in BCP ALL risk in children typing for *DP2* (GEG), a 98% increase in *DP2*-associated risk between 3 and 6 years of age, and a 130% increased risk associated with a single supergenotype, *DP2/DP4.* This association was not present in BCP ALL diagnosed <3 or >6 years of age, and leads us to conclude that the peak of BCP ALL (Greaves *et al*, 1993, 1985) may be influenced by the immunological sequelae of age-related interactions between *DP2/DP4* and a specific antigenic peptide derived from delayed infection.

Analysis of replicate case series, including the 128 BCP ALL cases new to this study, suggest strongly that the association with *DP2* was unlikely to be due to chance. Furthermore, *DP6*, which also has E at position 69 was not associated with BCP ALL, but was associated with non-BCP leukaemia. Phylogenetic analysis suggests that the *DPB1* peptide-binding motif may have undergone rapid recent diversification and *β69E* alleles, such as DPB1*0201 and DPB1*0601, are not all closely related (Gyllensten *et al*, 1996). Supertype analysis groups *HLA* alleles with convergent immunological properties (Hughes *et al*, 1996; Trachtenberg *et al*, 2003), based on common peptide-binding motifs, and may be more relevant to BCP ALL aetiology than individual alleles.

We measured the significance of case–control supertype frequency differences using Fisher's Exact tests, corrected for six supertypes or 21 supergenotypes. We did not correct for total *DP* alleles since our analysis was informed by the results of our previous study (Taylor *et al*, 2002) and would have been overly influenced by low frequency alleles. Nevertheless, our results require confirmation with independent case–control series.

Although associations between childhood ALL and *DR*, *DQ* and *DP* alleles have been reported in previous studies (Dorak *et al*, 1995, 1999; Taylor *et al*, 1995, 2002), there has been no test of the effect of LD between alleles at the different loci. We found no evidence that the association of BCP ALL with *DP2* could be explained by LD with *DQ* alleles, suggesting that *DP* has a primary role in susceptibility to BCP ALL.

It is unlikely that the association of BCP ALL with *DP2* is due to a defect in the immune response to an oncogenic virus (immune evasion). There is no evidence that childhood BCP ALL is caused by an oncogenic virus (MacKenzie *et al*, 2006), and the positive association with *DP2* suggests that binding of specific peptide(s) and T-cell activation are involved in causation, which is inconsistent with immune evasion by an oncogenic virus. The negative association of *DP1* with BCP ALL may be due to the binding and recognition of TEL-AML1 peptide(s) in children with pre-ALL with this supertype, as discussed elsewhere (Taylor *et al*, 2008), since a TEL-AML1 junctional peptide has been shown to elicit a DPB1*0501-restricted (*DP1*) CD4+ T cell response (Yun *et al*, 1999).

The delayed response to infection hypothesis for BCP ALL (Greaves, 2006) proposes that a child carrying an *in utero-*initiated preleukaemic clone is vulnerable to the development of leukaemia if it is insulated from infection during the early postnatal period, but exposed at a later age. We previously reported that the risk of BCP ALL was greater in DPB1*0201 heterozygotes than homozygotes (Taylor *et al*, 2002), suggesting that BCP ALL might be the rare ‘down-side’ of the advantage that MHC-heterozygosity confers on immune responses to infection. Although evolution of *HLA* allelic diversity is thought to favour heterozygotes (Takahata and Nei, 1990), a recent study suggests that this advantage may be allele-specific (Lipsitch *et al*, 2003). Our finding that only one (*DP2/DP4*) of 15 heterozygous supergenotypes (*GEG/GKG*) is associated with BCP ALL fits this model.

Using DPB1*0201 peptide-binding data and molecular modelling (Diaz *et al*, 2005), it is possible to make predictions about the amino acid anchors at P1, P4, and P6 of peptides binding to DP2. Pocket 4 of DP2 is deeper, more negatively charged than DP4 (Diaz *et al*, 2003), giving it a greater affinity for positively charged nonpolar aromatic residues, such as glutamine (Q), arginine (R), and lysine (K). Furthermore, glycine (G) makes pocket 1 (*β*84) and pocket 6 (*β*11) deep and hydrophilic, preferentially-binding hydrophobic and aromatic amino acids, notably phenylalanine (F), and tyrosine (Y) (Berretta *et al*, 2003; Diaz *et al*, 2003, 2005). This predicts that infectious peptides with an ^1^FXXKXFXXA/V^9^ motif (where X is unknown, and P9 can be A or V) are likely to bind to DP2.

In this context, Van Steensel-Moll *et al* (1986) reported a negative (protective) association between childhood ALL and infections in the first year of life, and Rosenbaum *et al* (2005) documented a weak negative association between childhood ALL and bronchiolitis and pneumonia. Roman *et al* (2007) found a slight deficit in lower respiratory tract infection in the first year of life of UKCCS ALL cases diagnosed at 2–5 years. Together these findings suggest that the immune response to RSV infection may be a factor in BCP ALL. RSV is a highly contagious, weakly pathogenic, but strongly immunogenic virus that is widely distributed in the childhood population (Handforth *et al*, 2000; McNamara and Smyth, 2002). The G protein of RSV elicits CD4+ T-cell responses (De Graaf *et al*, 2004; De Waal *et al*, 2004), the peptide ^162^D-N^179^ containing two overlapping T-cell epitopes, ^163^FHFEVFNFV^171^ and ^165^FEVFNFVPC^173^ that are restricted by DPB1*0401 (*DP4*), and DPB1*0201 (*DP2*) (De Graaf *et al*, 2004). Both peptides have F at P1 and P6 suggestive of binding to GEG (*DP2*) and GKG (*DP4*), consistent with the association of BCP ALL with *DP2/DP4*. While this conclusion is speculative it points to a need for detailed sero-epidemiological studies of RSV in BCP ALL.

## Figures and Tables

**Table 1:** Number of cases and controls in this study

	**Number**			**Male/female**
**Study group^a^**	**Total**	**Male**	**Female**	**ratio**
Leukaemia	895	492	403	1.22
BCP ALL	687	373	314	1.19
Non-BCP leukaemia	208	119	89	1.33
Pro-B ALL	26	7	19	0.37
T ALL	75	51	24	2.12
AML	107	61	46	1.33
Solid tumour	409	218	191	1.14
Newborn controls	864	436	428	1.01

a Leukaemias are classified as B cell precursor ALL (BCP ALL) and non-BCP acute leukaemia including Pro-B ALL, T ALL and AML.

**Table 2:** *DPB1* supertypes of *DPB1* alleles

***DPB1* supertype**	**Peptide-binding motif^a^**	***DPB1* alleles with this supertype**
*DP2*	GEG	0201, 0202, 3301, 4801
*DP4*	GKG	0401, 0402, 2301, 2401, 4901, 5101
*DP6*	LED	0601, 0901, 1001, 1301, 1701, 2101, 3001
*DP3*	LKD	0301, 1401, 2001, 2501, 2601, 3501
*DP8*	GED	0801, 1601, 1901
*DP1*	GKD	0101, 0501, 5001

a *DPB1* supertypes assigned from di-allelic amino acids at positions *β*_1_11 (G,L), *β*_1_69 (E,K) and *β*_1_84 (G,D).

**Table 3:** *DPB1* supertype frequency in childhood leukaemia and solid tumours compared with controls

	**Leukaemia**				**Solid tumour**				
***DPB1* supertype**	**%**	**OR**	**95% CI**	***P***	**%**	**OR**	**95% CI**	***P***	**Newborn controls %**
*DP2* (GEG)	10.3	1.6	1.2–2.0	0.0002^*^	7.3	1.1	0.8–1.5	0.6	6.8
*DP4* (GKG)	59.6	1.1	0.9–1.2	0.38	60.3	1.1	0.9–1.3	0.3	58.0
*DP6* (LED)	6.8	1.3	1.0–1.8	0.04	6.5	1.3	0.9–1.8	0.2	5.2
*DP3* (LKD)	12.0	0.9	0.7–1.1	0.29	12.8	0.9	0.8–1.2	0.8	13.2
*DP8* (GED)	1.4	2.9	1.4–6.3	0.006^*^	0.6	1.4	0.5–3.9	0.8	0.5
*DP1* (GKD)	6.9	0.6	0.5–0.7	<10^−4^^*^	8.6	0.8	0.6–1.1	0.09	11.1
Global *χ*^2^		<10^−6^^**^				0.04			
Number=		895				409			864

^*^Significant (*P*<0.05) after correction for six supertypes.

^**^Significant (*P*<0.05) in global *χ*^2^ (CLUMP) analysis.

**Table 4:** *DPB1* supertype frequency in BCP ALL and non-BCP leukaemia compared with controls

	**BCP ALL**				**Non-BCP leukaemia**			
***DPB1* supertype**	**%**	**OR**	**95% CI**	***P***	**%**	**OR**	**95% CI**	***P***
*DP2* (GEG)	10.8	1.7	1.3–2.1	<10^−4^^*^	8.4	1.3	0.9–1.9	0.3
*DP4* (GKG)	60.0	1.1	0.9–1.2	0.27	57.9	0.9	0.8–1.2	1.0
*DP6* (LED)	6.2	1.2	0.9–1.6	0.24	8.9	1.8	1.2–2.7	0.007^*^
*DP3* (LKD)	11.6	0.9	0.7–1.1	0.21	13.0	0.9	0.7–1.3	0.9
*DP6* (GED)	1.5	3.2	1.5–7.0	0.004^*^	1.0	2.2	0.7–6.6	0.4
*DP1* (GKD)	6.4	0.5	0.4–0.7	<10^−5^^*^	8.4	0.7	0.5–1.1	0.1
Global *χ*^2^			<10^−6^^**^				0.04^**^	
Number=			687				208	

^*^Significant after correction for six supertypes.

^**^Significant (*P*<0.05) in global *χ*^2^ (CLUMP) analysis.

**Table 5:** *DPB1* supertype-associated risk of BCP ALL in replicate series of cases compared with controls

	**Series 1^a^**			**Series 2**			**Series 3**			**Series 4**		
***DPB1* supertype**	**OR**	**CI**	***P***	**OR**	**CI**	***P***	**OR**	**CI**	***P***	**OR**	**CI**	***P***
*DP2*	1.6	1.2–2.1	0.001^*^	1.8	1.3–2.4	0.003^*^	1.6	1.1–2.1	0.007^*^	1.9	1.3–2.9	0.006^*^
*DP4*	1.1	0.9–1.3	0.19	1.1	0.9–1.3	0.23	1.05	0.9–1.3	0.59	0.9	0.8–1.3	0.99
*DP6*	1.3	0.9–1.7	0.17	1.2	0.8–1.7	0.48	1.3	0.9–1.8	0.26	1.02	0.6–1.8	0.99
*DP3*	0.9	0.7–1.1	0.41	0.9	0.7–1.1	0.33	0.9	0.7–1.1	0.34	0.7	0.5–1.1	0.14
*DP8*	3.0	1.3–6.8	0.01	3.1	1.3–7.6	0.02	3.4	1.4–8.2	0.01	4.4	1.6–12.5	0.03
*DP1*	0.5	0.4–0.7	<10^−4^^*^	0.5	0.3–0.7	<10^−3^^*^	0.6	0.5–0.9	0.008^*^	0.7	0.4–1.1	0.13
												
Number=	559			344			343			128		

a Series 1: see Taylor *et al* (2002); series 2: 50% of series 1; series 3: 215 cases from series 1+128 cases from series 4; series 4: new cases in this study.

^*^Significant (*P*<0.05) after correction for six supertypes.

**Table 6:** Risk of BCP ALL, non-BCP leukaemia and paediatric solid tumours associated with *DPB1* supergenotypes, compared with controls

	**BCP ALL**			**Non BCP leukaemia**			**Solid tumour**		
**Supergenotype**	**OR**	**95% CI**	***P***	**OR**	**95% CI**	***P***	**OR**	**95% CI**	***P***
*DP2/DP2* (GEG/GEG)	0.9	0.4–1.9	0.99	0.9	0.3–2.8	0.99	1.02	0.4–2.4	0.99
*DP2/DP4* (GEG/GKG)	2.1	1.5–2.9	0.00004^*^	1.6	0.9–2.7	0.14	1.1	0.7–1.8	0.7
*DP2/DP6* (GEG/LED)	2.5	1.0–5.8	0.06	2.5	0.8–7.7	0.3	1.5	0.5–4.5	0.6
*DP2/DP3* (GEG/LKD)	1.5	0.7–3.1	0.42	0.8	0.3–3.6	0.99	1.1	0.4–2.8	0.99
*DP2/DP8* (GEG/GED)	3.8	0.3–41.7	0.88	12.5	1.1–138.5	0.38	—	—	—
*DP2/DP1* (GEG/GKD)	1.1	0.5–2.7	0.96	0.6	0.1–7.9	0.99	0.9	0.3–2.6	0.99
*DP4/DP4* (GKG/GKG)	0.9	0.7–1.1	0.40	0.8	0.6–1.1	0.17	1.1	0.9–1.4	0.3
*DP4/DP6* (GKG/LED)	1.9	1.2–3.0	0.006	2.7	1.6–4.9	0.002^*^	1.7	1.0–2.9	0.06
*DP4/DP3* (GKG/LKD)	1.1	0.8–1.4	0.80	1.2	1.2–14.6	0.1	1.1	0.7–1.5	0.8
*DP4/DP8* (GKG/GED)	2.7	0.9–7.1	0.08	1.9	0.1–15.3	0.99	0.6	0.1–2.9	0.7
*DP4/DP1* (GKG/GKD)	0.8	0.6–1.2	0.40	0.9	0.6–2.0	0.8	0.6	0.4–0.9	0.04
*DP6/DP6* (LED/LED)	0.6	0.2–1.7	0.48	1.1	0.3–4.0	0.99	1.7	0.7–4.3	0.4
*DP6/DP3* (LED/LKD)	0.5	0.2–1.1	0.09	1.1	0.04–3.1	0.67	0.2	0.07–0.81	0.02
*DP6/DP8* (LED/GED)	—	—	—	—	—	—	6.3	0.6–70.2	0.6
*DP6/DP1* (LED/GKD)	0.8	0.3–2.3	0.89	2.2	0.7–6.7	0.37	0.9	0.3–2.8	0.99
*DP3/DP3* (LKD/LKD)	0.7	0.4–1.2	0.23	0.8	0.4–1.8	0.76	1.1	0.6–1.9	0.9
*DP3/DP8* (LKD/GED)	3.3	0.8–12.8	0.16	2.5	0.4–15.0	0.95	2.1	0.4–10.5	0.7
*DP3/DP1* (LKD/GKD)	0.5	0.2–1.2	0.15	1.6	0.5–3.9	0.85	1.2	0.5–2.7	0.8
*DP8/DP8* (GED/GED)	—	—	—	—	—	—	—	—	—
*DP8/DP1* (GED/GKD)	3.8	0.3–41.7	0.88	—	—	—	—	—	—
*DP1/DP1* (GKD/GKD)	0.2	0.1–0.5	0.00004^*^	0.4	0.1–1.06	0.06	0.8	0.4–1.5	0.6
Number=	687			208			409		
									

^*^Significant (*P*<0.05) after correction for 21 supergenotypes.
